# 

**Published:** 1896-12

**Authors:** 


					﻿Southern Medical Record.
A Monthly Journal of Medicine and Surgery.
Vpl. XXVI. ATLANTA, GA., DECEMBER, 1896.	No. XIl\
BERNARD WOLFF, M. D., Editor. LOUIS H. JONES, M. D., Business Manager.
Associate Editors:
A. W. GRIGGS, M. D.	WILLIS F. WFSTMORELAND, M. D.
j. McFadden gaston, μ. d.
Entered at the Post-Office at Atlanta, Ga., as Second-Class Matter.
The Foote & Davies Co., Atlanta, Ga.
				

